# Genome Sequence of *Pseudomonas aeruginosa* Strain LCT-PA41, with Changed Metabolism after Space Flight

**DOI:** 10.1128/genomeA.01124-13

**Published:** 2014-01-09

**Authors:** Chao Liu, Juan Hu, Xiangqun Fang, Duchao Zhang, De Chang, Junfeng Wang, Tianzhi Li, Yinhua Guo, Wenkui Dai, Changting Liu

**Affiliations:** Nanlou Respiratory Diseases Department, Chinese PLA General Hospital, Beijing, Chinaa; BGI-Shenzhen, Shenzhen, Chinab

## Abstract

To explore the effects of space flight on microorganisms, *Pseudomonas aeruginosa* ATCC 27853 was sent into orbit for 398 h on the spacecraft ShenZhou VIII. Here, we present the draft genome sequence of the *P. aeruginosa* strain LCT-PA41, determined after space flight.

## GENOME ANNOUNCEMENT

It has been reported that astronauts suffered a significant number of microbial infections, including acute respiratory infection, conjunctivitis, and dental infection (1). During the Apollo 13 mission, one crewmember also developed a urinary tract infection caused by *Pseudomonas aeruginosa* (2). To explore the effects of space flight on microorganisms, *Pseudomonas aeruginosa* ATCC 27853 was sent into orbit by ShenZhou VIII (3). After space flight, we selected one *P. aeruginosa* strain, LCT-PA41, based on Biolog’s powerful carbon source utilization technology.

To obtain the genomic DNA of *P. aeruginosa* LCT-PA41, two types with 350-bp and 6-kb sequence libraries were prepared and were sequenced by the Illumina Hiseq 2000 at BGI-Shenzhen (China). The read length was 90 bp for both libraries, from which 689 Mb and 345 Mb of high-quality data were generated, respectively. The disclosed draft genome sequence was assembled with 48 scaffolds (>500 bp) and 191 contigs. The total length of the assembly sequence was 6,887,679 bp, including 114,107 bp of unknown bases (gap), and the average GC content was 66.16%. The *N*_50_ and *N*_90_ of the assembled scaffolds were 3,913,163 bp and 311,742 bp, respectively. From the genome composition analysis results, we found that the genome contained 6,476 genes with an average length of 943 bp, and the total length of the genes was 6,108,396 bp, which makes up 88.69% of the genome. Additionally, we found 47 tRNAs with a total length of 3,707 bp, which makes up 0.0538% of the genome. In addition, 3 rRNAs and 24 sRNAs were also determined. Furthermore, 186 tandem repeats with a total length of 18,216 bp were found in the genome.

### Nucleotide sequence accession number.

The whole-genome sequence of *Pseudomonas aeruginosa* strain LCT-PA41 has been deposited at DDBJ/EMBL/GenBank under the accession number ATJU00000000. The version described in this paper is the first version.
